# Dextran sodium sulfate inhibits the activities of both polymerase and reverse transcriptase: lithium chloride purification, a rapid and efficient technique to purify RNA

**DOI:** 10.1186/1756-0500-6-360

**Published:** 2013-09-08

**Authors:** Emilie Viennois, Fengyuan Chen, Hamed Laroui, Mark T Baker, Didier Merlin

**Affiliations:** 1 Department of Biology, Center for Diagnostics and Therapeutics, Georgia State University, Atlanta GA 30303, USA; 2 Department of Gastroenterology, Shanghai Fifth People’s Hospital, Fudan University, 128 Ruili Road, 200240, Shanghai, People’s Republic of China; 3 Veterans Affairs Medical Center, Decatur GA 30033, USA

**Keywords:** DSS, DNA-polymerase, Reverse transcriptase, Lithium chloride, RNA

## Abstract

**Background:**

Dextran sodium sulfate (DSS) is commonly used in mouse studies to induce a very reproducible colitis that effectively mimics the clinical and histological features of human inflammatory bowel disease (IBD) patients, especially ulcerative colitis. However, the mechanisms of action of DSS remain poorly understood, and observations by our laboratory and other groups indicate that DSS contamination of colonic tissues from DSS-treated mice potently inhibits the quantitative reverse-transcription polymerase chain reaction (qRT-PCR) amplification of mRNA.

**Results:**

A prior study used poly-A-mediated mRNA purification to remove DSS from RNA extracts, but we herein report a second efficient and cost-effective approach to counteract this inhibition, using lithium chloride precipitation to entirely remove DSS from RNAs. We also explored how DSS interferes with qRT-PCR process, and we report for the first time that DSS can alter the binding of reverse transcriptase to previously primed RNA and specifically inhibits the enzymatic activities of reverse transcriptase and *Taq* polymerase *in vitro*. This likely explains why DSS-treated colonic RNA is not suitable to qRT-PCR amplification without a previous purification step.

**Conclusion:**

In summary, we provide a simple method to remove DSS from colonic RNAs, and we demonstrate for the first time that DSS can inhibit the activities of both polymerase and reverse transcriptase. In order to reliably analyze gene expression in the colonic mucosa of DSS-treated mice, the efficiency rate of qRT-PCR must be the same between all the different experimental groups, including the water-treated control group, suggesting that whatever the duration and the percentage of the DSS treatment, RNAs must be purified.

## Background

Dextran sodium sulfate (DSS), a sulfated polysaccharide, is commonly used to induce colitis in rodents [1,2]. Originally reported in 1985 by Ohkusa *et al*. who used DSS to induce colitis in hamsters [3], the DSS model was thereafter extrapolated to mice [4]. This chemical compound is now extensively used by investigators studying pathogenesis of colitis and factors affecting colitis. The main interest of DSS-induced experimental colitis is that the model can mimic the clinical and histological features of human inflammatory bowel disease (IBD), with ulcerative colitis (UC) characteristics [5]. Moreover, DSS-based studies using various therapeutic agents for human IBD show that DSS-induced colitis can be used as a relevant model for the translation of mouse induced colitis to human disease [6].

Colitis is induced by the addition of DSS to drinking water. Depending on the concentration, duration, and frequency of DSS administration, the animals may develop acute colitis, chronic colitis, or even colitis-induced dysplastic lesions when combined with azoxymethane (AOM) treatment [7-9]. When mice are given drinking water containing 3.5% DSS, the typical features of colitis appear on day 3 and are maximally expressed by day 7 [10]. Laroui *et al.*[11] suggested that the DSS associates with medium-chain length fatty acids (MCFAs), such as dodecanoate, in the colonic lumen. There, the colonic epithelium absorbs and partially metabolizes MCFAs, potentially explaining how the DSS enters epithelial cells [11]. In addition to being extensively used to induce intestinal inflammation in wild-type (WT) mice, this method also potently disturbs the mucosal barrier of mice that are genetically susceptible to develop colitis (DSS could induce and/or exacerbate the colitis) [12,13]. Therefore we can approximate that one third of the researchers studying colitis in mice are using the DSS model, hence the importance of overcoming the pitfalls linked to this model. Although the DSS model has been fully exploited for 20 years, the underlying mechanisms of DSS-induced colitis are not yet entirely understood.

In our laboratory, we observed that the contamination by DSS of colonic tissue inhibits quantitative reverse-transcription polymerase chain reaction (q-RT-PCR) amplification. This inhibitory effect of DSS was observed in a dose-dependent manner, as previously described by Kerr *et al*. [14], who suggested a poly-A-purification-based technique to remove DSS from RNA extracts. We developed another efficient and low-cost technique that uses lithium chloride to entirely remove DSS from the RNAs after their extraction from colonic tissue. This technique has been extensively used in our laboratory and by others [15-18], but has never been reported in detail. Here, we describe the mechanisms underlying DSS-mediated inhibition of q-RT-PCR and we provide a detailed description of this lithium chloride-based RNAs purification method.

## Results and discussion

### *In vivo* DSS exposure completely inhibits the qPCR process without altering RNA integrity

**Figure 1:**
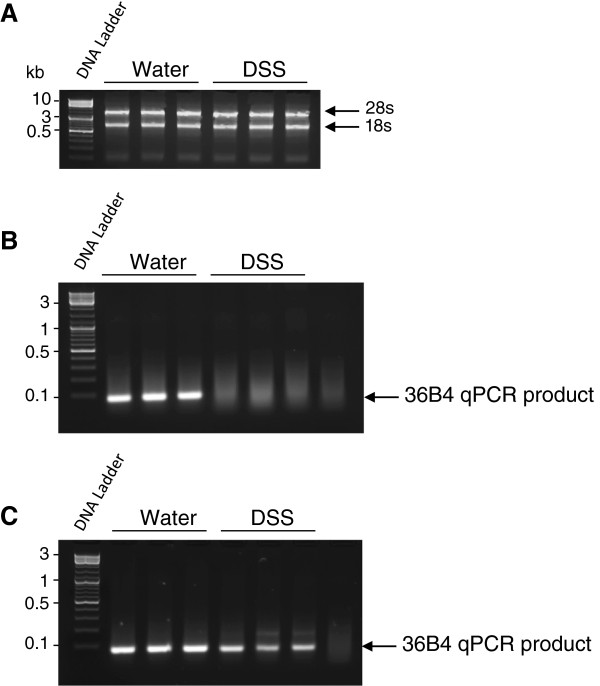
***In vivo *DSS exposure completely inhibits qPCR but does not alter RNA integrity. A)** Total RNA was extracted from colonic tissues obtained from mice treated with or without 3% DSS, and RNA integrity was assessed by 2% agarose gel electrophoresis. DSS treatment does not alter the integrity of the RNA. **B)** After cDNA synthesis, qPCR was performed for the 36B4 amplicon. The amplification of 36B4 was totally inhibited in DSS-treated mice. **C)** The DSS- and non-DSS RNA samples were purified using our lithium chloride protocol, and qPCR was performed. After lithium chloride purification, 36B4 was successfully amplified from both DSS- and non-DSS samples.

To test whether *in vivo* DSS exposure can alter RNA integrity, WT mice were exposed to drinking water containing 3% DSS for 7 days. RNAs from colon tissues of control (water-treated) and DSS-treated mice were extracted using the TRIzol reagent and resolved by electrophoresis. RNA samples from control and DSS-treated colons showed perfect integrity (Figure 1A), indicating that DSS treatment does not alter RNAs synthesis and/or stability. Next, cDNA synthesis and qPCR were performed on total RNA from control and DSS-treated colons. Agarose gel electrophoresis revealed that housekeeping gene 36B4 amplification products were obtained from control samples but not from DSS-treated samples (Figure 1B), demonstrating that the *in vivo* exposure of colonic tissue to DSS result in a complete inhibition of qPCR process. We speculated that this could arise *via* inhibition of cDNA synthesis and/or general inhibition of the qPCR process itself.

### Purification of RNAs using lithium chloride abolish the inhibitory effect of DSS on qPCR

To remove all polysaccharides (including DSS) from the samples, we purified the RNA using our lithium chloride protocol (for details, see Methods). Briefly, the RNA were precipitated twice by 0.1 volume of 8 M LiCl, followed by a precipitation step in 0.1 volume of 3 M sodium acetate (pH 5.2) and 2 volumes of 100% ethanol. The RNA were then centrifuged, pellets were washed with 100 μL of 70% ethanol and RNAs were finally dissolved in 20-50 μl of RNase-free water. After this purification step, 36B4 amplification products were observed from both DSS-treated and control samples (Figure 1C), indicating that this simple purification protocol is a very powerful tool to remove DSS from RNA samples and counteract the previously observed inhibition of qPCR process. These data also confirm that the cDNA synthesis and/or qPCR reactions cannot occur properly in the presence of DSS. To exclude the possibility that DSS directly binds to RNA, we performed surface plasmon resonance (SPR) experiments with increasing concentrations of DSS. RNAs were coupled to the gold sensor surface of the chip, and increasing concentrations of DSS were flowed over the RNA-coupled chip surface. Our results indicated that DSS molecule does not bind to RNA (Additional file 1).

### Inhibition of the qPCR process does not occur with individual components of DSS

**Figure 2:**
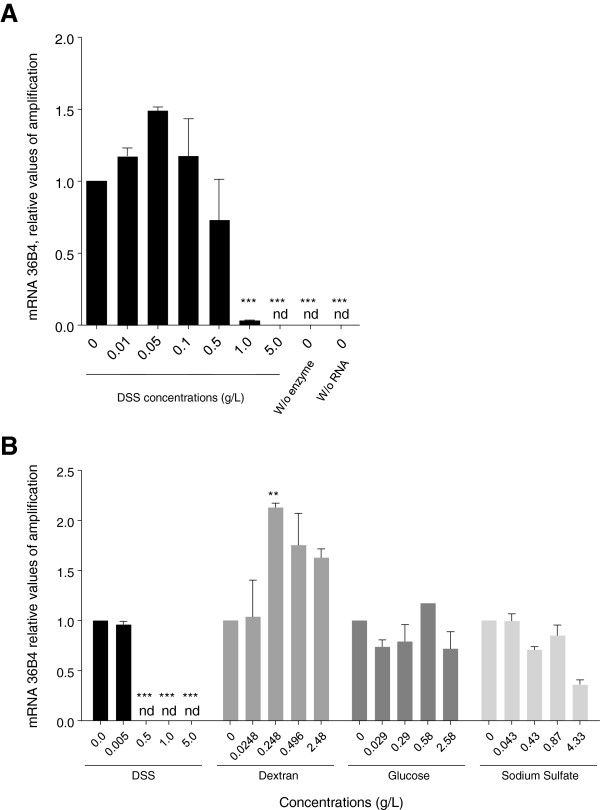
***In vitro *RNA exposure to DSS, but not DSS-associated molecules, completely inhibits qPCR. A)** Total RNA from non-DSS samples was incubated *in vitro* with different concentrations of DSS, and the qPCR amplification of 36B4 was assessed. The amplification of 36B4 was blocked by 1 or 5 g/L of DSS. Abbreviations: ***, p < 0.001; nd, not detected. **B)** Total RNA from non-DSS samples was incubated *in vitro* with different concentrations of DSS or its components (dextran, glucose and sodium sulfate), and the qPCR amplification of 36B4 was assessed. The amplification of 36B4 was fully blocked by DSS, but the component compounds did not alter the amplification of 36B4 at any tested concentration. Abbreviation: **, p < 0.01; ***, p < 0.001; nd, not detected.

*In vitro* experiments were used to examine the mechanism by which the DSS inhibits qPCR amplification. Colonic RNA from water-treated mice were treated *in vitro* with increasing concentrations of DSS, and then subjected to cDNA synthesis and qPCR. Treatment with low concentrations of DSS (0.01 to 0.5 g/L) did not significantly alter 36B4 amplification, whereas concentrations of DSS over 0.5 g/L completely inhibit 36B4 amplification (Figure 2A). Since DSS is composed of successive glucose units substituted with sulfur groups, we next investigated the effect of each DSS component (dextran, glucose, and sodium sulfate) at concentrations based on those of the DSS solution. In one polymer chain of DSS, 138 glycosyl residues were present. Equivalent concentrations for DSS associated molecules have been calculated considering the equivalent number of glucose or sulfate motifs, according to Laroui *et al.*[11]. Our results revealed that increasing concentrations of dextran, glucose, or sodium sulfate do not alter the amplification of 36B4 (Figure 2B), indicating that the inhibitory effect observed is specific to the entire DSS molecule.

### DSS inhibits the activity of *Taq* polymerase

**Figure 3:**
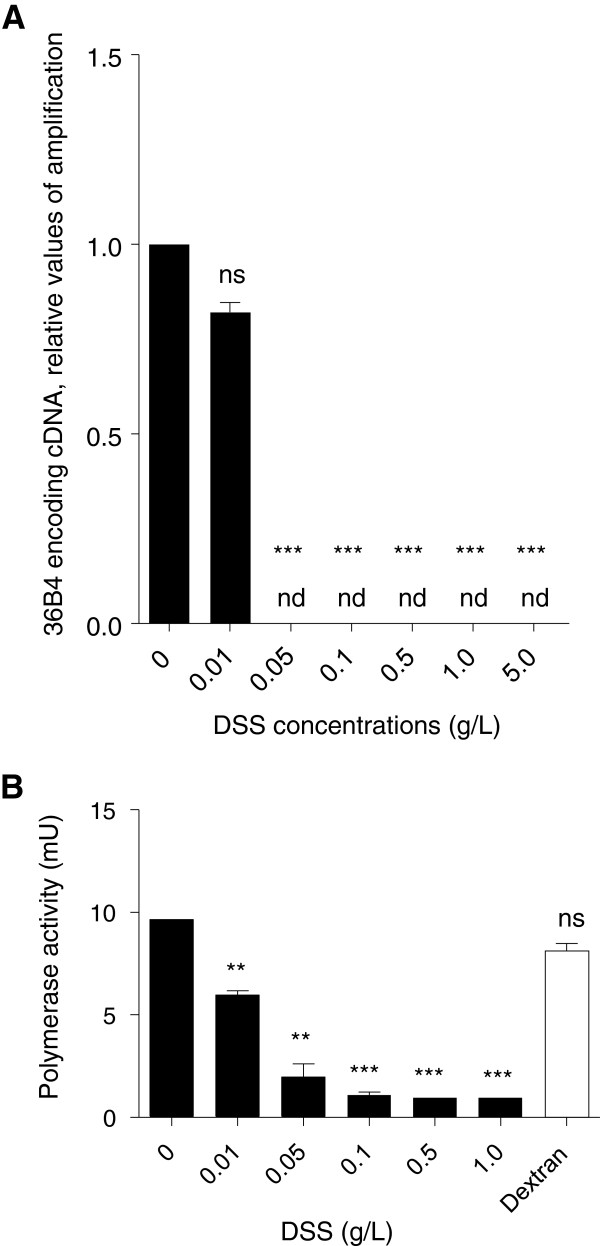
**DSS inhibits the activity of *Taq *polymerase. A)** RNA from non-DSS-treated tissue was reverse transcribed, the cDNA was incubated *in vitro* with DSS, and the amplification of 36B4 was assessed by qPCR. Incubation with 0.01 g/L DSS triggered a small but significant decrease in the amplification of 36B4, and 0.05 g/L and higher concentrations of DSS fully inhibited the amplification of 36B4 by qPCR. Abbreviations: ns, non-significant; nd, not detected; ***, p < 0.001. **B)** Constant amounts of *Taq* polymerase (10 mU) were incubated with water or increasing concentration of DSS, and polymerase activity was assessed. DSS dose-dependently decreased the polymerase activity. Abbreviations: **, p < 0.01; ***, p < 0.001.

To test whether DSS inhibits *Taq* polymerase activity, we synthesized cDNA from total RNA obtained from non-DSS-exposed colonic tissue, incubated it with increasing concentrations of DSS after the reverse transcription step, and subject them to PCR amplification. Our results revealed that DSS strongly inhibited PCR amplification at concentrations over 0.01 g/L. (Figure 3A). To test if this reflect inhibition of the *Taq* polymerase itself, we incubated a constant quantity of *Taq* polymerase with different concentrations of DSS, and used an enzyme assay kit to perform fluorescence-based quantification of polymerase activity. Results revealed that DSS dose-dependently decreased the polymerase activity (Figure 3B). In contrast, the polymerase activity was not altered in the presence of dextran alone (a component of DSS). These findings demonstrate that DSS specifically and dose-dependently inhibits *Taq* polymerase activity.

### DSS inhibits the activity of the reverse transcriptase

**Figure 4:**
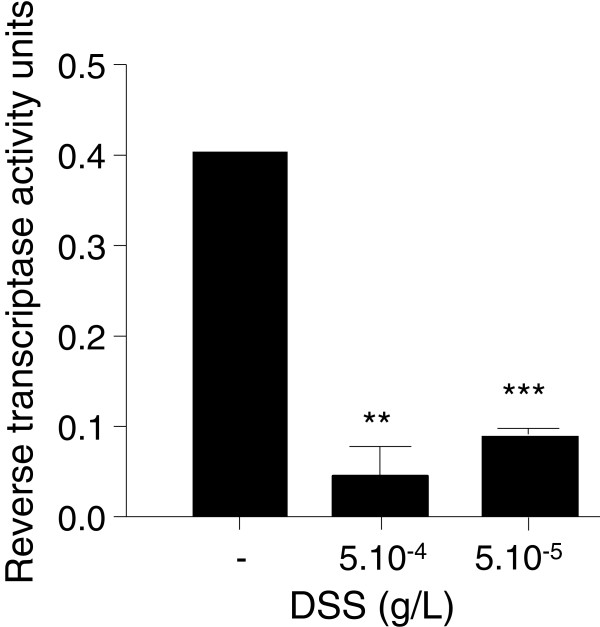
**DSS inhibits the activity of the reverse transcriptase.** Constant amounts of M-MuLV reverse transcriptase (0.4 U) were incubated with water or DSS, and reverse transcriptase activity was assessed. DSS decreased the activity of the reverse transcriptase. Abbreviations: **, p < 0.01; ***, p < 0.001; ns, non-significant.

To assess whether DSS also inhibits the reverse transcriptase activity, we incubated a constant amount of reverse transcriptase (0.4 U) with DSS or water and assessed reverse transcriptase activity using an enzyme assay kit. Results indicate that the reverse transcriptase activity is drastically decreased in the presence of 5.10^-4^ or 5.10^-5^ g/L of DSS, compared to water control (Figure 4). Thus, DSS appears to block the RT-qPCR process by inhibiting both *Taq* polymerase and reverse transcriptase.

### DSS alters the interaction between primed RNAs and reverse transcriptase

**Figure 5:**
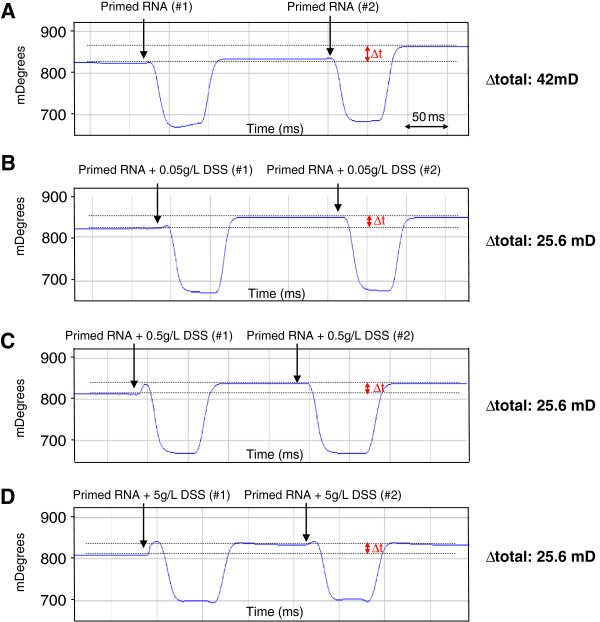
**DSS alters the interaction between primed RNA and reverse transcriptase.** Primed RNA was incubated with or without different concentrations of DSS, and binding to the M-MuLV reverse transcriptase was determined by SPR (measured in mDeg). **A)** The primed RNA binds to the reverse transcriptase, deflecting the resonance angle to 42 mDeg. **B-D)** After incubation with 0.05 **(B)**, 0.5 **(C)** or 5 **(D)** g/L of DSS, the primed RNA binds to the reverse transcriptase, deflecting the resonance angle to 25.6 mDeg. Δt = Δtotal.

To examine the mechanism by which the DSS alters reverse transcriptase activity, we used SPR to analyze whether DSS modifies the interaction between reverse transcriptase and primed RNA. Two successive injections of 2,400 units of M-MuLV Reverse Transcriptase were performed to load the enzyme on the chip. A quantity of 830 ng of RNA that had been pre-primed with random primers was injected twice as the analyte. We found that after two injections, the primed RNA directly bound to the reverse transcriptase, deflecting the resonance angle to 42 mDeg (Figure 5A). The experiment was then repeated with primed RNA that had been pre-incubated with different concentrations of DSS (0.05, 0.5 and 5 g/L). Interestingly, the presence of DSS decreased the binding of primed RNA to the reverse transcriptase by 1.6 fold and deflected the resonance angle to 25.6 mDeg, regardless of the concentration (Figure 5B-D).

The injection of various concentrations of DSS alone did not deflect the resonance angle, demonstrating that DSS alone did not directly bind to the purified reverse transcriptase (data not shown). Taken together, these results indicate that DSS alters the binding between reverse transcriptase and primed RNA, partly explaining the inhibitory effect of DSS on reverse transcriptase activity.

## Conclusion

DSS is widely used to induce experimental colitis. Our lab and others have found that DSS contamination of colonic tissue potently inhibits the RT-qPCR process [14,16]. Thus, qPCR-based analysis of mRNA expression in DSS-exposed tissues requires removal of the contaminating DSS. To address this, we developed an efficient and low-cost lithium chloride-based technique to entirely remove DSS from RNAs. In this article, we provide a simple protocol based on lithium chloride purification (for details, see Methods) that will allow researchers to analyze the effect of DSS-induced colitis on gene expression levels.

We also report here *in vitro* experiments investigating the mechanism by which DSS inhibits qPCR. Our results revealed that DSS does not bind to RNA or alter its integrity, but rather inhibits the activity of both reverse transcriptase and DNA-dependent Taq polymerase. This inhibition is specific to DSS, as its component molecules (glucose, dextran and sodium sulfate) did not inhibit RT-qPCR. In addition, we found that the inhibitory effect of DSS on reverse transcriptase activity is at least partially due to its ability to alter the binding between RNAs and the reverse transcriptase.

DSS has been shown to interact with various cellular and bacterial components, thereby altering certain biological mechanisms. For example, DSS could competes with poly(U) [19] and inhibits ribonuclease activity [20,21]. Other natural and synthetic polyanionic polymers have been found to play important roles in the association of mRNA with ribosomes, and thus in mRNA translation [19]. Together with our data, these reports suggest that DSS can interact with cell components and alter the replication process.

Based on observations that DSS can inhibit qPCR, we herein sought to elucidate the underlying mechanism. Our novel findings showing that DSS inhibits the activity of both reverse transcriptase and *Taq* polymerase increase our understanding of how DSS may act *in vivo* and suggest that further studies are warranted to examine the impact of this commonly used experimental reagent on whole-animal physiology, reverse transcriptase and polymerase activities.

## Methods

### DSS treated mice

All studies were performed in accordance with the Institutional Animal Care and Use Committee at Georgia State University (Atlanta, GA). All procedures were approved and are registered in the protocol IACUC ID: A11025, approval date 8/30/2011 to 8/30/2014. Strains, ages, and the number of animals follow the established protocol.

The DSS treatment on mice were carried out in C57BL/6 mice (8 wk, 18–22 g) obtained from Jackson Laboratories (Bar Harbor, ME). Mice were group housed under a controlled temperature (25°C) and photoperiod (12:12-h light–dark cycle) and allowed unrestricted access to standard mouse chow and tap water. DSS [40,000 Da, 3% (wt/vol), ICN Biochemicals, Aurora, OH] was diluted at 3% in drinking water. After 7 days under DSS treatment, the mice were sacrificed by CO_2_ euthanasia. A small piece (50 mg) of proximal colon was taken for RNA extraction.

### RNA extraction

Total RNA was isolated from colonic tissues using TRIzol (Life Technologies, Carlsbad, CA) according to the manufacturer’s instructions. Where indicated, RNA was purified *via* precipitation with lithium chloride. The RNA integrity was assessed by 2% agarose gel electrophoresis.

### Lithium chloride purification

In order to be purified from all polysaccharides including DSS, a purification using lithium chloride was performed. The RNA were incubated with 0.1 volume of 8 M LiCl diluted in RNase-free water on ice for 2 h and then centrifuged at 14,000 g for 30 min at 4°C. The supernatants were poured out and the pellets of RNA dissolved in 200 μl of RNase-free water. The 2-hour incubation with lithium chloride, the centrifugation and the pellet suspensions were repeated once more. The RNA was precipitated at −20°C for 30 min, in 0.1 volume of 3 M sodium acetate (pH 5.2) and 2 volumes of 100% absolute ethanol. The RNA was then centrifuged at 14,000 g for 30 min at 4°C. The supernatants were poured out and the pellets were washed with 100 μL of 70% ethanol and centrifuged at 14,000 g for 10 min at 4°C. The supernatants were removed and the RNA was dissolved in 20–50 μl of RNAse-free water.

### cDNA synthesis and qPCR

cDNA were synthesized using the Maxima First-Strand cDNA Synthesis Kit (Thermo Scientific, Waltham, MA) according to the manufacturer’s instructions. Expression of the total RNA was quantified by qPCR using Maxima SYBR Green/ROX qPCR Master Mix (Thermo Scientific) in a Realplex Thermal Cycler (Eppendorf, Hauppauge, NY). The qPCR primer sequences for 36B4 were 36B4-F: TCCAGGCTTTGGGCATCA and 36B4-R: CTTTATCAGCTGCACATCACTCAGA.

### Polymerase assay

The polymerase enzymatic activity was measured using the EvaEZ™ Fluorometric Polymerase Activity Assay kit (Biotium, Hayward, CA) according to the manufacturer’s instructions. Briefly, 0.01 units (10 mU) of recombinant *Taq* DNA polymerase (Thermo Scientific) were incubated with water or DSS (0.01 to 1 g/L). Dextran was used as a control. The enzymatic activity was quantified by fluorescence using the Realplex Thermal Cycler (Eppendorf). The fluorescence was read every 1 min for 60 min during the elongation step at 72°C.

### Reverse transcriptase assay

The reverse transcriptase enzymatic activity was measured using the EnzChek Reverse Transcriptase Assay kit (Life Technologies, Carlsbad, CA) according to the manufacturer’s instructions. Briefly, 0.4 units of the reverse transcriptase, M-MuLV RT (Thermo Scientific) were incubated at 25°C with water or DSS (0.00005 g/L or 0.0005 g/L) in presence of a standardized polyA RNA template, oligodT and polymerization buffer. The enzymatic activity was quantified by fluorescence using a Synergy 2 Multi-Mode Microplate Reader (Biotek).

### Surface plasmon resonance (SPR)

For SPR experiments, gold sensor chips were used (Biosensing Instrument, Tempe, AZ, USA). Briefly, the principle of this technique is the following: A first molecule is coupled to the gold sensor surface. The solution containing the second molecule (the analyte) then is flowed over the surface. This creates a mass change on the sensor surface as the two molecules interact, which is detected in real time as a deflection of the resonance angle in mDeg. In that specific experiment, the gold chip was cleaned and treated as previously described [22-24]. After placing a chip into the BI-2000 SPR (Biosensing Instrument) machine each gold biosensor chip covered with carboxydextran was activated using a mixture of 1-ethyl-3-(3-dimethylaminopropyl) carbodiimide/N-hydroxysuccinimide (EDC/NHS) to form amide linkages between purified protein and the chip-bound carboxydextran. Two successive injections of 2,400 units of M-MuLV Reverse Transcriptase (thermo Scientific) each were performed. The reverse transcriptase was previously purified using Slide-A-Lyser® Mini dialysis devices (ThermoScientific) and suspended in PBS. After coating the chip with reverse transcriptase, RNA, previously primed using random primers (Thermo Scientific) incubated at 42°C, or primed RNA incubated with DSS in different concentrations (0.05, 0.5, 5 g/L) were passed over the chip twice. A two-step interaction curve was obtained. The first step involved adsorption of primed RNA to the maximal level. In the second step, when the flow of primed RNA concentration returned to zero, nonspecific adsorbed primed RNA were released with the running buffer. The deviation of the resonance angle thus decreased to a plateau located at a level above the initial baseline. We assessed the laser deflection as directly correlated to the binding level. We thus used the laser deviation angle as the optimal parameter for the binding affinity. All comparisons between the different solutions of primed RNA and primed RNA with different concentrations of DSS were performed as a measure of the laser deviation in mDegrees (mDeg).

### Statistical analysis

Values were expressed as means ± standard error of mean (SEM). Statistical analysis was performed using an unpaired two-tailed t-test by GraphPad Prism 5 software. p <0.05 was considered statistically significant.

## Availability of supporting data

The data sets supporting the results of this article are included within the article and its additional file.

## Abbreviations

AOM: Azoxymethane; CD: Crohn’s disease; DSS: Dextran sodium sulfate; IBD: Inflammatory bowel disease; LiCl: Lithium chloride; SPR: Surface plasmon resonance; UC: Ulcerative colitis.

## Competing interests

The authors declare that they have no competing interests.

## Authors’ contributions

EV and DM conceived and designed the experiments, analyzed the data, and wrote the manuscript. EV performed the experiments. MTB edited the manuscript. FC contributed reagents and materials. HL provided input into the project’s direction. All authors read and approved the final manuscript.

## Supplementary Material

Additional file 1 **RNAs were coated to the gold chip.** The binding of DSS to the RNA was determined by SPR (measured in mDeg). Increasing concentrations of DSS (0.2, 0.3, 0.4, 0.5 and 1 g/L) were passed over the chip. No deflection of the laser angle was observed meaning the DSS does not bind to the RNA. Click here for file
