# Supplementary figures and images for: Dextran sodium sulfate inhibits the activities of both polymerase and reverse transcriptase: lithium chloride purification, a rapid and efficient technique to purify RNA
Source: BMC Res Notes. 2013 Sep 8;6:360. doi: 10.1186/1756-0500-6-360 (PMC3847706; doi:10.1186/1756-0500-6-360)

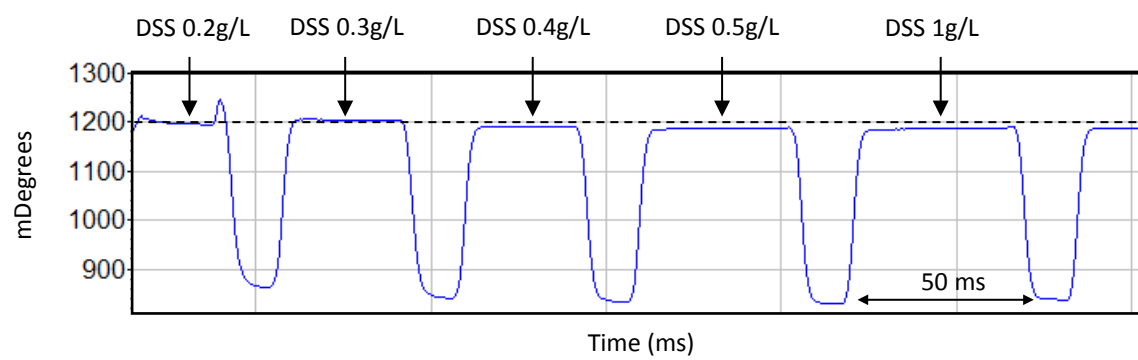

Supplement: Additional file 1 — RNAs were coated to the gold chip. The binding of DSS to the RNA was determined by SPR (measured in mDeg). Increasing concentrations of DSS (0.2, 0.3, 0.4, 0.5 and 1 g/L) were passed over the chip. No deflection of the laser angle was observed meaning the DSS does not bind to the RNA. [file 1756-0500-6-360-S1.pdf]
